# Effects of group cognitive behavior therapy on cognitive flexibility in college students with high obsessive-compulsive symptoms

**DOI:** 10.3389/fpsyg.2025.1527933

**Published:** 2025-08-12

**Authors:** Sifan Ji, Yingying Liu, Lu Chen, Wei Zhang, Kongliang He

**Affiliations:** ^1^School of Mental Health and Psychological Sciences, Anhui Medical University, Hefei, China; ^2^Affiliated Psychological Hospital of Anhui Medical University, Hefei Fourth People’s Hospital, Hefei, China; ^3^Anhui Mental Health Center, Hefei Fourth People’s Hospital, Hefei, China; ^4^Psychiatry Department, Hefei Fourth People’s Hospital, Hefei, China; ^5^Psychological Counseling Department, Hefei Fourth People’s Hospital, Hefei, China

**Keywords:** group cognitive behavior therapy (GCBT), cognitive flexibility, obsessive-compulsive symptoms, college students, psychological intervention

## Abstract

**Objective:**

To explore the effect of group cognitive behavior therapy on cognitive flexibility in college students with high obsessive-compulsive symptoms.

**Methods:**

Fifty-eight college students were randomly divided into an experimental group and control group (29 in each group). The experimental group was treated with group cognitive behavior therapy (twice a week for 4 weeks) whereas the control group was untreated. Before and after the intervention, the two groups of students were tested with the obsessive-compulsive scale OCI-R, cognitive flexibility scale CFI, acceptance and action questionnaire AAQ-II, and the cognitive fusion questionnaire CFQ to evaluate the effect after the intervention.

**Results:**

In the total score of obsessive-compulsive scale, the interaction between measurement time and groups was significant (*F*(1,56) = 17.563, *p* < 0.001). The post-test score of the experimental group was significantly lower than that of the pre-test score, and there was no significant difference in the control group before and after the intervention. In the total score of the cognitive flexibility scale, the interaction between measurement time and groups was significant (*F*(1,56) = 6.616, *p* < 0.05). The post-test score of the experimental group was significantly higher than the pre-test score, and there was no significant difference in the control group before and after the intervention. In the second edition of the action questionnaire and the cognitive fusion questionnaire, the interaction between measurement time and groups was significant (*F*(1,56) = 18.887, *p* < 0.001). The post-test score of the experimental group was significantly lower than that of the pre-test, and there was no significant difference in the control group before and after the intervention.

**Conclusion:**

Taken together, our results show that group cognitive-behavioral therapy significantly improved the cognitive flexibility of college students with high obsessive-compulsive symptoms, resulting in a significant reduction of these symptoms.

## 1 Introduction

Cognitive flexibility is the ability to adjust behavior and needs flexibly in response to changing environmental demands (Armbruster et al., 2012). It is an important component of behavioral cognitive control (Banich, 2009) and encompasses a broad spectrum of human abilities, including the recognition of and adaptation to various situational needs. It plays a significant role in personal development, interpersonal communication, and environmental adaptation (Ionescu, 2012; Johnco et al., 2014). Many researchers believe that cognitive flexibility is a prerequisite for many mental functions and among the most important factors affecting intelligence and creativity (Diamond, 2013). Research suggests that cognitive flexibility is compromised in individuals with obsessive-compulsive disorder (OCD) and those exhibiting high OCD traits, resulting in psychological rigidity and subsequent cognitive impairment (García-Villamisar and Dattilo, 2015). A suicidal behavior model suggests that when cognitively rigid individuals are under high stress conditions, they are not cognitively prepared with effective alternative solutions to deal with stressors in their environment, placing such individuals at high risk for suicidal behavior (Schotte and Clum, 1982).

The clinical manifestation of OCD is compulsive thinking, the repeated occurrence of compulsive behavior, or both. The prevalence of OCD in China is 2.5–3.0% (Huang et al., 2019). Obsessive-compulsive disorder and obsessive-compulsive symptoms are two different concepts. While the former constitutes the core symptomatology in patients, not all individuals with obsessive-compulsive symptoms meet the full diagnostic criteria for OCD (Gellatly et al., 2017). College students face a heightened risk of experiencing obsessive-compulsive symptoms. Hou et al. investigated 4,119 college students, finding that 22.1% exhibited obsessive-compulsive symptoms (Hou et al., 2018). Liu Guohua screened 15,000 college students and found that 12.4% had compulsive symptoms (Liu, 2017). Compulsive symptoms have emerged as a common psychological problem among college students (Doolub et al., 2023). Obsessive-compulsive symptoms may lead to executive and cognitive deficits. This can also result in general discomfort in daily interpersonal relationships and difficulty responding flexibly (Gruner and Pittenger, 2017). The disorder carries a high disability rate, often leading to lifelong illness and a decline in the quality of life of both patients and their families, thereby impacting social functioning (Huz et al., 2016).

Cognitive behavioral therapy (CBT) is considered the most effective form of psychotherapy based on evidence-based medicine to treat OCD (Ost et al., 2015; Koran et al., 2007; Hunsley et al., 2014). The efficacy of CBT for OCD has been demonstrated in randomized controlled trials (Kathmann et al., 2022). Between the classical CBT model and acceptance and commitment therapy (ACT), we chose a CBT model that placed more emphasis on changing irrational beliefs and behavioral activation. ACT focuses on accepting a bad experience rather than subjectively trying to change it. ACT pays attention to the relationship between behavioral factors and cognitive factors that may exist in the treatment of patients, to help patients face the formation of their mentality through psychological intervention more objectively. As such, patients with low self-esteem, tension, anxiety, and other negative psychological have seen significantly alleviated symptoms and improved compliance with the treatment.

Cognitive flexibility encompasses various aspects, such as cognitive thinking rigidity, inflexibility, behavioral challenges, and environmental adaptation difficulties. CBT posits that rigid thinking is a significant factor contributing to abnormal mood or behavior (Kazantzis et al., 2018). College students possess a higher cognitive level, better treatment compliance, and are more suitable for the classic CBT mode. Both case-specific and group-based CBT are considered effective treatments for OCD (Rogers et al., 2017; Wootton, 2016) and the first recommended treatment for newly diagnosed patients. The most effective case-specific CBT treatment is exposure and response prevention. However, group CBT may be a more suitable and effective method for college students (Hirschtritt et al., 2017). Students with high compulsive symptoms and poor cognitive flexibility can better feel the power of the group and adapt to interpersonal communication problems.

There is extensive intervention research on OCD in China, although relatively little has focused specifically on cognitive flexibility in college students with severe OCD symptoms. Another pressing issue is that the resources of psychological counselors in colleges and universities are limited. This makes it difficult for many college students who suffer from obsessive-compulsive symptoms to receive timely and effective psychological assistance. In view of these resource constraints, group psychological counseling is an efficient and low-cost psychological treatment method, showing potential as an effective way to mitigate the effects of OCD in college students.

To improve the cognitive flexibility of college students with high obsessive-compulsive symptoms, this study was guided by CBT combined with related theories of group counseling to design a group intervention plan based on obsessions, impaired cognitive flexibility, and their characteristics, and to explore whether group CBT can improve the cognitive flexibility of college students with high obsessive-compulsive symptoms.

## 2 Research object

### 2.1 Research object

The whole group was sampled from two colleges and universities in Hefei, China, taking the class as a unit. A total of 754 questionnaires were issued in this survey. Of these, 699 were recovered and 12 invalid questionnaires were excluded, leaving 687 valid questionnaires (recovery efficiency: 91.1%). The mean age was 19.55 ± 1.00 years, with 260 males (37.8%) and 427 females (62.2%).

### 2.2 Criteria for inclusion

(1) Total OCI-R score >29, included in the high obsessive symptoms group;
(2) Age 16 years or older.

Exclusion criteria:

(1) Exclusion of mental disorders;
(2)Patients with serious physical diseases;
(3)Those who did not want to participate in the group cognitive behavioral therapy activities and those who could not be guaranteed to participate in the treatment activities for 4 weeks.

A total of 60 college students in the high obsessive symptoms group were selected. Two were excluded from the group cognitive behavioral therapy intervention through interviews, so that 58 were ultimately enrolled. Twenty-nine subjects each were randomly assigned to the experimental group and control group. They were 19.86 ± 0.99 years old and had received 12.97 ± 0.325 years of education. The control group consisted of subjects who were aged 20.14 ± 0.99 years that had received 13.1 ± 0.489 years of education. There was no significant difference in test age (*t* = −1.061, *p* = 0.293) or years of education (*t* = −1.265, *p* = 0.211) between the experimental group and the control group.

### 2.3 Measures

#### 2.3.1 Compulsive scale revision (OCI-R)

This scale was simplified and revised by Foa et al. (2002) based on the 1998 version. The Chinese version was revised by Tang et al. (2011). The scale has six dimensions: washing, obsessing, hoarding, ordering, checking, and mentai neutralization. In this study, a scale with an internal consistency coefficient of 0.88 was used to assess the intensity of compulsive symptoms in patients.

#### 2.3.2 Cognitive flexibility inventory (CFI)

The CFI was developed by Dennis and Vander Wal (2009). A revised Chinese version was also developed (Wang et al., 2016). There are 20 CFI topics, divided into two dimensions: selectivity and controllability. The higher the score, the higher the flexibility. The internal consistency coefficient, selectivity, and controllability dimensions of the total questionnaire in this study were 0.88, 0.85, and 0.83, respectively.

#### 2.3.3 Acceptance and action questionnaire-II (AAQ-II)

The AAQ-II scale was modified by Cao et al. (2013) and contains seven items, each of which is scored at 7 levels. The higher the score, the higher the degree of empirical avoidance, the higher the degree of psychological rigidity, and the worse the cognitive flexibility. The internal consistency coefficient of the scale was 0.88.

#### 2.3.4 Cognitive fusion questionnaire (CFQ)

The Cognitive Fusion Questionnaire is a Chinese version co-edited by Zhang et al. (2014). The internal consistency coefficient was 0.92. Its intrinsic coefficient of consistency is 0.92. The higher the score of this scale, the higher the degree of cognitive integration, the higher the degree of psychological rigidity, and the lower the cognitive flexibility.

### 2.4 Group cognitive behavioral therapy program

#### 2.4.1 Group leader

Main leader: responsible for the design and preparation of the group plan in the activity, leading the activity. Cooperate with the leader: give feedback and suggestions on the implementation of the activity plan in the activity and assist the smooth progress of the activity. The group was a homogeneous and closed group. After each intervention, the group leaders were supervised and discussed the key points and problems encountered during the intervention. Two licensed clinical psychologists (5 + years CBT experience) led sessions, with weekly supervision by the last author (a CBT specialist). Fidelity was ensured via manual adherence checks (90% compliance).

#### 2.4.2 Group program

The design of the group counseling program was mainly based on CBT, group psychological counseling and counseling-related theories, interpersonal communication, and other theories. It combined the characteristics of compulsive symptoms, cognitive rigidity, and the actual situation and environment of college students. The program consisted of eight themes, each of which was 90 min activity conducted twice a week for 4 weeks. In the process of intervention, we followed a series of characteristics shown by students: confused and irritable emotions for university life, flexible adaptation to changes in the university environment and interpersonal relationships, and the pursuit of a perfect self. The intervention measures were designed with the main idea of identification, acceptance, changing unreasonable beliefs, and a flexible response. First, the therapy aimed to help subjects objectively understand negative emotions, cognitive rigidity, and unreasonable beliefs, and to reduce the internal consumption of mental energy caused by confrontation with them. Then, subjects aimed at self-understanding and self-acceptance were provided and the group explored various coping styles and perspectives. Finally, the participants were asked to share their feelings, review their perceptions of their behavior, and to think flexibly through the process and practice and apply the lessons in real life while dealing with parting emotions (Table 1).

**Table 1:** Treatment activity plan.

Activities	Activity content	Activity goal
1. Meet	Group leaders introduce themselves; make personal business cards; sign informed consent; poetic and artistic; initial cognitive flexibility; personal expectation; share the experience of the activity; assign homework	Get to know each other and build trust; establish group norms; understanding cognitive theory
2. Feel emotion	Review homework; learn to name emotions; three-column table learning; sharing experience activities; assign homework	Strengthen the acceptance of each other among team members; learn to recognize emotions; learn to evaluate emotions; three column table learning
3. Recognize automatic emotions	Review homework; building block puzzle games; introduction to automatic thinking; mental distortion checklist exercise; discussion of coping styles; three-column table learning; share the experience of the activity; assign homework	Learning to recognize automatic thinking; learn to use the cognitive three-column chart; learn to recognize irrational beliefs
4. Correcting false beliefs	Review homework; guess the numbers; methods of correcting unreasonable beliefs; five-column table learning; share the experience of the activity; assign homework	Clarify individual irrational belief and correct unreasonable cognition; learn to use five-column tables
5. Meet different possibilities, different angles	Review homework; our school; secret meeting - brainstorming; five column table exercises; share the experience of the activity; assign homework	Look at the problem from different angles and propose multi-angle solutions to improve the selectivity of cognitive flexibility; exercise to deepen the five-column table
6. Make the impossible possible - Respond positively	Review homework; classic slogan; five column table exercises; share the experience of the activity; assign homework	Guide members to face difficulties with more confidence; increased controllability of cognitive flexibility
7. Self-management behavior activation	Review homework; auction life; life pie chart; share the experience of the activity; assign homework	Encourage each other to increase confidence; clear time management allocation, improve self-worth; looking into the future, shaping new behavior patterns; increased self-control
8. Summarize	Review homework; 20 years from now; advantages bombing; reflection and summary; send blessings; end group	Summarize the group; farewell group

## 3 Research design and analysis

### 3.1 Study design and evaluation

We adopted a mixed design method of 2 (group) × 2 (time) for the design and evaluation. ‘Group’ was considered to an intergroup variable that included two levels of experimental and control groups, where ‘measurement time’ is an intra-group variable that includes two levels: pre - and post-intervention. The experimental group was required to participate in group counseling for four consecutive weeks, while the control group did not. All participants filled out questionnaires before and after the test. All participants signed a confidentiality agreement and informed consent prior to the intervention.

### 3.2 Statistical analysis

IBM SPSS 27.0 software was used to analyze the data. *T*-tests and variance analysis were used to conduct statistical analysis of the questionnaire. *p* < 0.05 was considered statistically significant.

## 4 Results

### 4.1 Comparison of differences in obsessive-compulsive symptoms between the experimental group and control group

After 4 weeks of intervention, ANOVA showed that the interaction between the measurement time and group was significant in the total value of the obsessive-compulsive symptom scale: *F*(1,56) = 17.563, *p* < 0.001. Simple-effect analysis showed that there was no significant difference between the two groups. The post-measured value of the experimental group was significantly lower than the pre-measured value. That is, the total score of obsessive-compulsive symptoms was significantly reduced after intervention (*T* = 5.9, *p* < 0.001). Before intervention, there was no significant difference between the experimental group and the control group.

The interaction between time and group was significant in the dimensions of obsessive-compulsive concepts, hoarding, ordering, and psychological neutralization (*F*(1,56) = 18.496, *p* < 0.01; *F*(1,56) = 13.25, *p* < 0.01; *F*(1,56) = 8.055, *p* < 0.05; *F*(1,56) = 4.462, *p* < 0.05; and *F*(1,56) = 4.971, *p* < 0.05, respectively). The interaction was not significant in the washing dimension. Simple-effect analysis showed that there was no significant difference between the control group before and after measurement. After intervention, the experimental group showed significant improvements in obsessive-compulsiveness, hoarding, ordering, and examination (*T*-values: 5.791, 5.86, 4.612, and 3.946, respectively; all *p* < 0.001); and significantly decreased psychological scores (*T* = 3.763, *p* < 0.01). In the dimension of washing, the scores of the experimental group also decreased significantly after intervention (*T* = 2.719, *p* < 0.05) (see Table 2).

**Table 2:** Descriptive statistics of variables in the experimental group and the control group (*M* ± SD).

Variable	Experimental group (*n* = 29)		Control group (*n* = 29)	
	Before measurement	After test	Before measurement	After test
OCI-R total points	37.24 ± 6.334	25.69 ± 7.969^**^	34.00 ± 6.601	31.83 ± 7.112
Washing	4.45 ± 2.08	3.21 ± 1.78^*^	4.48 ± 1.993	4.17 ± 1.891
Obsessing	7.21 ± 2.094	4.72 ± 1.791^**^	6.00 ± 2.563	5.76 ± 2.081
Hoarding	7.22 ± 1.688	5.41 ± 2.323^**^	6.97 ± 1.955	6.62 ± 1.935
Ordering	6.48 ± 1.785	4.69 ± 1.692^**^	5.97 ± 1.973	5.55 ± 2.131
Checking	6.31 ± 2.301	4.55 ± 2.261^**^	6.21 ± 2.289	5.76 ± 2.047
Neutralizing	5.07 ± 2.034	3.31 ± 2.089^*^	4.34 ± 1.818	3.90 ± 1.676
CFI total points	65.07 ± 5.988	69.00 ± 6.541^**^	66.52 ± 9.661	66.48 ± 9.383
Choosability	42.72 ± 4.728	43.59 ± 4.800	43.38 ± 6.748	43.28 ± 6.546
Controllability	22.34 ± 2.931	25.41 ± 2.946^**^	23.14 ± 3.729	23.21 ± 3.688
AAQ-II	27.97 ± 4.881	21.52 ± 4.725^**^	25.48 ± 4.672	24.14 ± 5.43
CFQ	38.52 ± 5.89	30.21 ± 6.538^**^	35.86 ± 6.39	34.07 ± 6.75

*p < 0.05, **p < 0.01.

### 4.2 Comparison of differences in cognitive flexibility between the experimental group and control group

The interaction between the time and group was significant in terms of total CFI score, *F*(1,56) = 6.616, *p* = 0.016. Simple-effect analysis showed that the difference between the pre- and post-tests in the control group was not significant, and the scores of the experimental group after intervention significantly improved compared with the pre-test group (*T* = −3.081, *T* = 0.005). In the two dimensions of selectivity and controllability, the interaction between selectivity time and group was not obvious, but the interaction between controllability time and group was obvious. Simple-effect analysis showed that there was no significant difference between the pre- and post-tests in the control group, and the scores of the experimental group significantly improved after the intervention in controllability (*T* = −5.532, *p* < 0.001), although there was no significant improvement in selectivity. In the AAQ-II total score, the interaction between time and group was significant, *F*(1,56) = 18.887, *p* < 0.001. Simple-effect analysis showed that there was no significant difference in the test before and after the control group, but the test group had a significant decrease in the test score (*T* = 7.279, *p* < 0.001). In the total CFQ score, the interaction between time and group was significant, *F*(1,56) = 13.129, *p* = 0.001. Simple-effect analysis showed that there was no significant difference before and after the test in the control group, and the score of the experimental group was significantly lower than that of the pre-test (*T* = 5.965, *p* < 0.001) (see Table 2).

### 4.3 Correlation analysis of cognitive flexibility level and obsessive-compulsive symptoms in experimental group and control group after intervention

Pearson correlation analysis was performed on the scores for obsessive-compulsive symptoms and cognitive flexibility of the experimental group and control group after intervention. The results showed that the total score of CFI was negatively correlated with the total score of OCI-R (*r* = −0.197), and that the subscale controllability was significantly negatively correlated with it (*r* = −0.339**). There was a significant positive correlation between AAQ-II and OCI-R total score (*r* = 0.466**). There was a significantly positive correlation between CFQ and the total score of OCI-R (*r* = 0.526* *) (see Table 3).

**Table 3:** Correlation analysis between obsessive-compulsive symptoms and cognitive flexibility scores in the experimental group and the control group after intervention (*n* = 58).

Variable	CFI total point	Selectivity	Controllability	AAQ-II	CFQ
OCI-R	−0.197	−0.073	−0.339**	0.466**	0.526**
Washing	−0.277*	−0.223	−0.281*	0.239	0.314*
Obsessing	−0.265*	−0.114	−0.429**	0.605**	0.646**
Hoarding	−0.130	−0.066	−0.195	0.325*	0.424**
Ordering	0.029	0.118	−0.126	0.076	0.165
Checking	−0.045	0.027	−0.149	0.223	0.226
Neutralizing	−0.059	0.008	−0.150	0.389**	0.343**

*p < 0.05, **p < 0.01.

## 5 Discussion

This study found that CBT-based group counseling can effectively improve the cognitive flexibility of college students with high obsessive-compulsive symptoms. On the obsessive-compulsive symptom scale, the total score of the experimental group decreased significantly after intervention compared with the control group. We also found significant improvements in different dimensions such as obsessive-compulsive thinking, hoarding, sequencing, examination, and psychological neutralization, indicating that CBT group counseling can improve obsessive-compulsive symptoms in a more comprehensive and obvious way. Our results are consistent with those of other studies (Rogers et al., 2017; Wootton, 2016). We conducted Pearson correlation analysis on obsessive-compulsive symptom scores and the cognitive flexibility level of the experimental group and control group after intervention. The results showed that after group cognitive behavioral therapy, the cognitive flexibility of the students significantly improved and their obsessive-compulsive symptoms were alleviated. We found that with the improvement of obsessive-compulsive symptoms, there was a corresponding increase in controllability and decrease in cognitive rigidity. The increased level of cognitive flexibility significantly improved compulsive symptoms.

In terms of the total score and controllability dimension of the cognitive flexibility scale, the scores of the experimental group significantly improved after intervention. There was no difference in the control group. These results indicate that the cognitive flexibility of the subjects improved, unreasonable beliefs had been corrected to some extent, and their sense of certainty and control of cognition had been enhanced. On the AAQ-II scale and CFQ scale, the scores of the experimental group decreased significantly, while there was no significant difference in the control group, indicating that the degree of cognitive rigidity in the experimental group reduced and the level of cognitive flexibility improved.

On the CFI subscale, the improvement of controllability was more obvious, and the improvement of selectivity was not obvious. Selectivity here has two meanings: the awareness that there are multiple explanations for the causes of life events and behaviors; and the acceptance of a variety of ways to deal with difficulties. This indicates that interventions may have a less selective impact on specific problems in the short term and are less helpful for solving difficulties in multiple ways. Subjects are more likely to pay attention to controllability and reduce uncertainty. Controllability refers to an individual’s ability to recognize that a difficult situation is controllable. It has been found that perfectionism (reducing uncertainty) is an important factor affecting OCD (Sun et al., 2009). Therefore, on this scale, subjects will pay more attention to whether they are more confident at completing a task and determine to a greater extent that difficult situations can be controlled.

It is generally believed that individuals with higher cognitive flexibility have more positive psychosomatic states and more positive coping styles (Dennis and Vander Wal, 2009). Learners with high cognitive flexibility assessed by numeral-letter switching tasks perform better at probabilistic rule tasks, indicating that cognitive flexibility is correlated with performance after practicing the rules of probabilistic rule tasks (Feng et al., 2020). Other studies have shown that cognitive flexibility plays an intermediary role in the optimism and happiness of college students, and there is a positive correlation between cognitive flexibility and school adaptation (Demirtaş, 2020). Cognitive flexibility is associated with the use of various thinking strategies and mental frameworks. People with cognitive flexibility have the ability to investigate the environment to identify changes that occur and develop multiple strategies to prepare for any changes that may occur (Calarco and Gurvis, 2006). The intervention in this study effectively changed the cognitive flexibility of the group members. First, members felt a safe atmosphere and established a sense of security, for example by signing informed consent and group commitments at the beginning stage. Second, members also had a more objective understanding of obsessive-compulsive symptoms and cognitive flexibility, understanding negative emotions and irrational beliefs, and reducing their mental consumption. Third, members learned to look at themselves from multiple perspectives in the group atmosphere, especially in the process of “secret meeting - brainstorming.” Members shared their feelings and said that they gained a lot and accepted themselves to a greater extent. Finally, members were actively involved in actions and a vision for the future. At the end of the group, we conducted “strength bombing” to summarize the feelings of the whole process and discuss the parting emotions. This encouraged them to have a new understanding of their symptoms, of themselves, and of life, to better adapt to life, to take a multi-angle view of problems, and to reduce the habit of thinking in set ways with cognitive rigidity.

## 6 Limitations and prospects

This study has several limitations that should be acknowledged. First, the relatively small sample size may limit the generalizability of the findings and reduce statistical power to detect significant effects. Second, the absence of a control or comparison group makes it difficult to attribute observed outcomes solely to the intervention, as confounding variables may have influenced the results. Third, the lack of follow-up assessments prevents an evaluation of whether the intervention’s effects were sustained over time. Finally, the group-based nature of the intervention introduces the possibility of placebo or nonspecific effects, such as social support or group dynamics, which could have contributed to the observed changes independent of the intervention itself. Future research should address these limitations by employing larger, more diverse samples, incorporating control conditions, and including longitudinal follow-ups to assess long-term efficacy. Individuals with low cognitive flexibility often suffer from various mental health problems, such as depression, anorexia, anxiety disorder, and unipolar affective disorder (Calarco and Gurvis, 2006; Moore and Fresco, 2007). Therefore, we can use the feature of cognitive flexibility to understand the mental health of students with other characteristics. Although there is increasing attention on the psychological problems of college students, the mental health problems of graduate and doctoral students are becoming more and more prominent. Future studies could expand the sample to include these students. Group cognitive behavioral therapy can deal with negative emotions and general psychological problems quickly and efficiently. The treatment is generally easy to implement in colleges and universities and doing so can prevent and promote mental health.

## Data Availability

The raw data supporting the conclusions of this article will be made available by the authors, without undue reservation.
